# Sagliker Syndrome in a Patient With Secondary Hyperparathyroidism and Chronic Kidney Disease: A Case Report From Palestine

**DOI:** 10.7759/cureus.51956

**Published:** 2024-01-09

**Authors:** Ibtihal Ahmad, Saja Alkomi, Rula Sharaha, Shaheera Manasrah, Osama N Dukmak

**Affiliations:** 1 Nephrology, Internal Medicine, School of Medicine, Palestine Polytechnic University, Hebron, PSE; 2 Nephrology, Internal Medicine, School of Medicine, Al-Quds University, Jerusalem, PSE

**Keywords:** case report, secondary hyperparathyroidism, renal osteodystrophy, chronic kidney disease, sagliker syndrome

## Abstract

Sagliker syndrome (SS) is a rare complication in patients with chronic kidney disease (CKD) on prolonged dialysis due to uncontrolled secondary hyperparathyroidism (SHPT). SS manifests with a constellation of clinical manifestations, including short stature, craniomaxillofacial abnormalities, hearing loss, and neuropsychiatric disorders. This article reports a 33-year-old male patient with CKD who complained of progressive disfiguring facial changes, multiple recurrent fractures, and shortened height. The condition affects his quality of life. On workup, his lab results showed highly elevated serum levels of parathyroid hormone, alkaline phosphatase (ALP), calcium, and phosphate. His comorbidities and poor health status limit his ability to do parathyroidectomy (Ptx). A reliable diagnostic approach must be considered, enabling physicians to make earlier interventions and get better outcomes.

## Introduction

In 1864, Virchow first coined the term leontiasis ossea to describe a condition in which patients have facial bone enlargement [1]. Over the years, it was realized that leontiasis ossea is not a disease by itself but comes in association with other conditions such as Paget’s disease, gigantism, and craniofacial fibrous dysplasia. Later in 1953, Cohen and Diamond were the first ones to describe skull deformity in the craniomaxillofacial region in patients with end-stage renal disease (ESRD) who developed secondary hyperparathyroidism (SHPT) resulting in lion-like face appearance, with names like uremic leontiasis ossea, bighead, or swollen face [2]. This phenomenon was first defined in detail by Sagliker and his colleagues in 2004 and the incidence is approximately 0.5% in patients on hemodialysis [3].

Sagliker syndrome (SS) is a devastating form of renal osteodystrophy resulting from untreated or inadequately treated SHPT. Acquired short stature is the most common change noticed in patients with SS. The craniofacial features of patients with SS are characterized by irregular alterations in skull and facial bones, causing patients to look very similar to thalassemic faces. These changes are typically seen in maxillary, mandibular, and nasal bones. Oropharyngeal abnormalities manifest as irregularly shaped and located teeth, tongue and soft tissue hyperplasia in the upper oral cavity, and class II malocclusion of the maxillary bones [4]. Other features include a retracted nasal bridge, upward curved fingertips, knee and scapular deformities, hearing loss, and severe neuropsychiatric disorders [5].

The exact etiology of SS is unknown, but several studies suggest that it is a multifactorial condition. In approximately half of the patients, inheritable mutations were established. There is a relation between chromosome aberrations (CAs) and SS progression. In addition, a missense mutation on the GNAS1 gene contributes to its pathogenesis [6]. Another predisposing factor of SS induction is the high alkaline phosphatase (ALP) and intact parathyroid hormone (iPTH) levels. The high level of PTH secondary to hyperphosphatemia, active vitamin D deficiency, anemia, and hypocalcemia contributes to hyperplasia and hypertrophy of the parathyroid gland cells in ESRD [7].

In 2016, a study in Bulgaria involving four patients who met the criteria for SS concluded that loss of control of blood levels of calcium and phosphorus in patients with chronic kidney disease (CKD) could lead to severe bone changes and psychological changes in patients with prolonged dialysis. SS is not well-known worldwide, and a few cases are reported in the literature. We report a 33-year-old male with CKD, severe SHPT, and clinical findings of SS. Our study is the first study in Palestine. Thus, we aim to draw more attention, increase awareness about this novel condition, and encourage the medical field to make early interventions to stop the disease progression.

## Case presentation

The patient was a 33-year-old male with a history of ESRD and poorly controlled hyperparathyroidism. He had been on hemodialysis for 11 years, beginning at the age of 22 years but with poor compliance and did not attend his regular follow-up visits.

**Figure 1:**
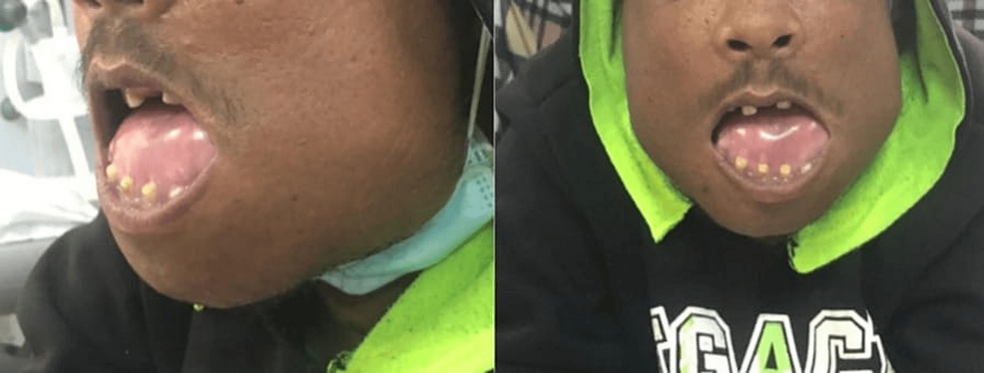
Unique facial features include maxillary and mandibular overgrowth, malocclusion of the upper and lower jaws, irregularly wide-spaced teeth, and puffy tongue

**Figure 2:**
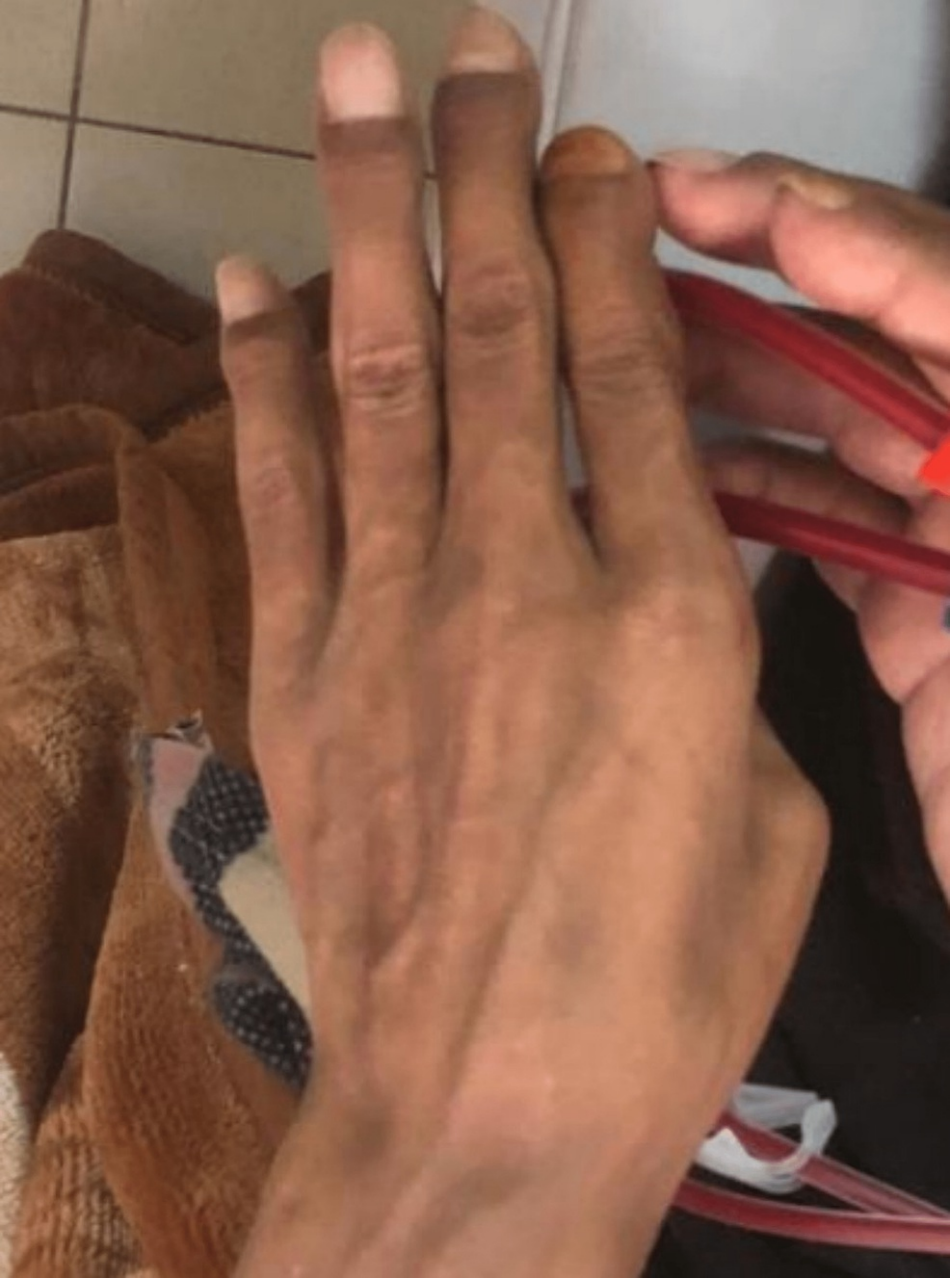
Upward curved fingertips

**Figure 3:**
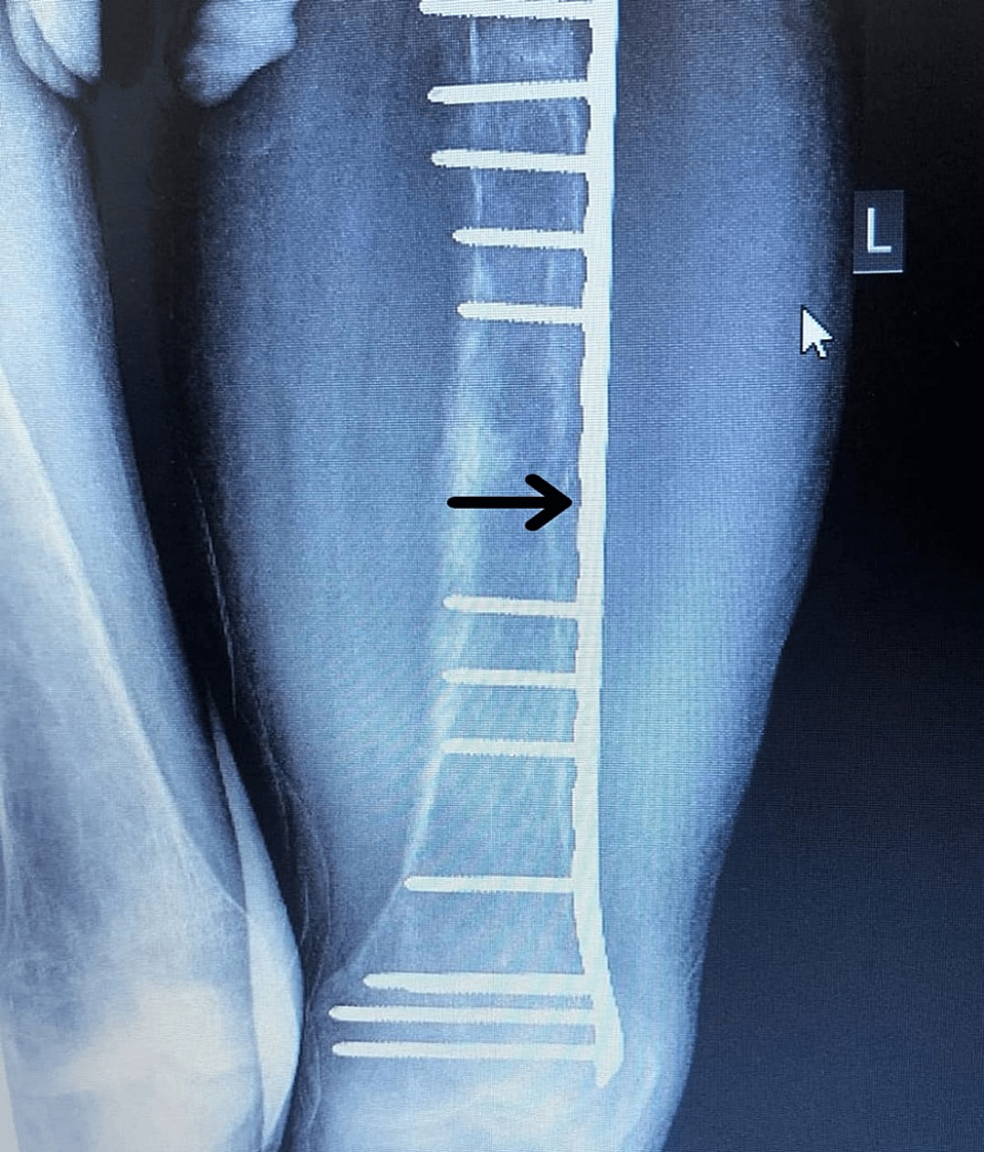
Open reduction and internal fixation (ORIF)

**Figure 4:**
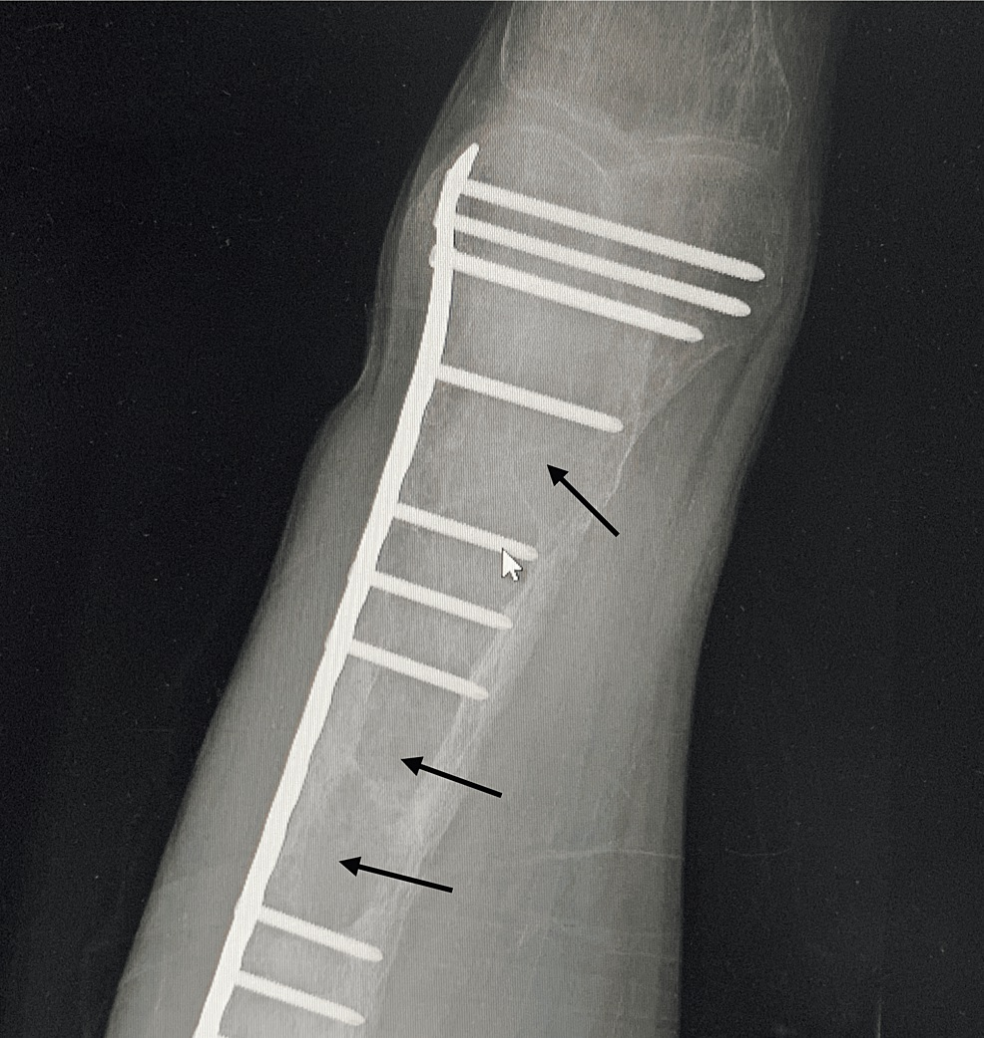
Multiple osteitis fibrosa cystica

The patient noticed significant progressive changes in his facial appearance over the last ten years, including bilateral symmetrical maxillary and mandibular protrusion, depressed nasal bridge, irregularly wide-spaced teeth, and a puffy tongue (Figure 1). These changes lead to difficulty chewing and swallowing and dysphonia. His fingertips became curved upward as well (Figure 2). Also, he mentioned a history of recurrent fractures in his extremities with minor trauma that required surgical fixation, caused by multiple osteitis fibrosa cystica secondary to severe hyperparathyroidism (Figure 3 and Figure 4). His physical activity had deteriorated as he had experienced recurrent falls. Ultimately, he had become wheelchair-bound and unable to care for himself. He reported that his height shortened by 15 cm over the past few years. His PTH levels had been severely elevated at >2500 pg/mL (normal range: 10-80 pg/mL). Over the years, he had developed weakness and bone pain in all four extremities, especially severe leg pain while walking. He had hearing loss as well.

With the overall poor health status of the patient, including poorly controlled hypertension, decompensated heart failure, poor compliance with dialysis therapy, and delayed presentation, doctors were weighing the risks for the benefits. They concluded that he was unable to undergo surgery. His complaints lead to a psychological impairment affecting his quality of life and the ability to communicate with others; he becomes socially withdrawn, does not get out of the home without wearing a face mask, and has an irritable mood most of the time.

Table 1 shows the historical lab measurements of PTH, serum calcium level, phosphorus, and ALP over the recent years.

**Table 1:** Historical lab measurements PTH, parathyroid hormone; ALP, alkaline phosphatase; NA, not available

	2018	2019	2021	2022	2023
PTH	>2500	2500 pg/mL	3500 pg/mL	3000 pg/mL	NA
ALP	1690 U/L	170 U/L	1826 U/L	1805 U/L	1223 U/L
Serum calcium	15 mg/dL	10.2 mg/dL	9.5 mg/dL	8.7 mg/dL	8 mg/dL
Phosphorus	4.26	NA	7.2	4.7	3.7

## Discussion

Despite the universal changes in the phosphate-calcium metabolism that occurs in CKD and the development of SHPT in 40% of patients, SS is a unique, serious complication of uncontrolled SHPT corresponding to a subgroup of patients with CKD [8]. The clinical manifestations of this syndrome include severe maxillary or mandibular deformities and benign oral tumors. The mechanism of craniofacial bone overgrowth is unclear but could be explained by intramembranous ossification activation in the craniomaxillofacial region [9,10].

In addition to dental abnormalities, other symptoms include fingertip changes, neuropsychiatric disturbances, short stature, and severe orthopedic changes such as scapular deformities, x- or o-type knee deformities, and crippled walking in particularly left legs [5]. Also, hearing loss is reported in 60% of the patients [11].

SS is more common in developing countries due to monetary deficiencies, the inability to access optimal CKD therapy, or iatrogenic mistreatment [11]. Although the etiology is still unclear, several risk factors have been identified, including female gender, high serum phosphate levels, prolonged duration of dialysis, and genetic mutations in the calcium-sensing receptor (CaSR) gene exons 2 and 3 and exons 1, 4, and 10 of the GNAS1 gene, GNAS1 gene, FGF23, and FGFR3 genes [8,10,12].

Concerning biochemical markers, the serum levels of iPTH and ALP in the SS group compared with a control group of those who only have SHPT and CKD were significantly higher (P<0.001), while the levels of serum calcium and phosphorus were not hugely different between the two groups [10].

Over the past years, efforts on a large scale to enhance the treatment of SHPT and suppress its catastrophic outcomes were taken. The treatment includes medical and surgical options. Medical therapy involves, for example, calcimimetic agents that work by slowing or stopping the progression of bone changes. If the optimized medical option fails to provide adequate regulation, surgery is the next option. There are three types of surgeries, including total parathyroidectomy (tPtx), tPtx with autotransplantation (tPtx+AT), and subtotal Ptx (sPtx) [10].

Unfortunately, these procedures are associated with a high recurrence rate of SHPT [13-14]. Thus, the patients need long-term follow-up to monitor for recurrences and evaluate their quality of life, especially those with one of these risk factors PTH level (>106.5 pg/mL) on the first postoperative day, phosphorus level (>5.9 mg/dL) on the first postoperative day, and starting time from dialysis to primary Ptx (≤3 years) [10,15]. Yet there are no clearly established guidelines for the management of recurrent SHPT. However, patients can be followed according to KDIGO guidelines 2009 or Japanese Society for Dialysis Therapy 2012 guidelines, which set a target serum iPTH target range of two to nine times the upper normal range. The KDIGO guidelines state that patients with CKD stage five should check their serum iPTH level every three months and serum calcium every four to six hours for the first two to three days after surgery and then twice daily after stabilization.

The recurrent SHPT may arise from the neck, mediastinum, or autografted forearm. The causative parathyroid glands could be localized using imaging modalities such as US, CT, MIBI scintigraphy, or magnetic resonance imaging (MRI) [16]. Reversal of severe skeletal deformities is impossible, while better control of SHPT may stop its progression and provide a better quality of life [8].

## Conclusions

SS is a rare disorder that may be encountered in CKD patients with inappropriately treated or uncontrolled SHPT. Poor control of calcium and phosphorus levels in the blood in CKD patients also plays a role, which leads to severe changes and deformities in bone, craniofacial abnormalities, and psychological impairment.

The delayed presentation may significantly impact the patient's life, so appropriate management must begin as early as possible in specialized centers. Regular checkups for iPTH, ALP, Ca, and phosphorus serum levels can lead to earlier detection and intervention and better outcomes. We should take more steps to build a reliable approach to diagnosis.
